# Genome Sequence of *Maribius* sp. Strain MOLA 401, a Marine *Roseobacter* with a Quorum-Sensing Cell-Dependent Physiology

**DOI:** 10.1128/genomeA.00997-14

**Published:** 2014-10-02

**Authors:** Margot Doberva, Sophie Sanchez-Ferandin, Yoan Ferandin, Laurent Intertaglia, Fabien Joux, Philippe Lebaron, Raphaël Lami

**Affiliations:** a Sorbonne Universités, UPMC Université Paris, USR 3579, LBBM, Observatoire Océanologique, Banyuls-sur-Mer, France; b CNRS, USR3579, LBBM, Observatoire Océanologique, Banyuls-sur-Mer, France; c Sorbonne Universités, UPMC Université Paris, UMR7232, BIOM, Observatoire Océanologique, Banyuls-sur-Mer, France; d CNRS, UMR7232, BIOM, Observatoire Océanologique, Banyuls-sur-Mer, France; e Sorbonne Universités, UPMC Université Paris, UMS 2348, Observatoire Océanologique, Banyuls-sur-Mer, France; f CNRS, UMS2348, Observatoire Océanologique, Banyuls-sur-Mer, France; g Sorbonne Universités, UPMC Université Paris, UMR 7621, LOMIC, Observatoire Océanologique, Banyuls-sur-Mer, France; h CNRS, UMR 7621, LOMIC, Observatoire Océanologique, Banyuls-sur-Mer, France

## Abstract

*Maribius* sp. strain MOLA401 is an alphaproteobacterium isolated from a coral reef lagoon located in New Caledonia, France. We report the genome sequence and its annotation which, interestingly, reveals the presence of genes involved in quorum sensing. This is the first report of a full genome within the genus *Maribius*.

## GENOME ANNOUNCEMENT

*Maribius* sp. strain MOLA401 (MOLA culture collection WDCM911 [http://collection.obs-banyuls.fr/index.php]) was isolated on 3 December 2004, at a 4-m depth, from marine waters in the southwest lagoon of New Caledonia (France) (22°21.23′ S/166°23.43′ E). The sampling station was located between oligotrophic water near the coral barrier and mesotrophic waters in the near-shore environment subjected to terrestrial inputs and effluents from the city of Nouméa (146,000 inhabitants) (1). Based on its 16S rRNA gene sequences, the strain is phylogenetically related to *Maribius pelagius* B5-6^T^ (96% sequence identity of 16S rRNA genes) and belongs to the *Rhodobacteraceae* (*Roseobacter* clade).

The strain was cultivated in 100 mL of marine broth 2216 medium (BD Difco, Sparks, MD) at 25°C for 48 h. DNA was extracted using a cetyltrimethylammonium bromide (CTAB)-based method (2). Genome sequencing steps conducted by MrDNA (Texas) included fragmentation, ligation to sequencing adapters, and purification. Following the amplification and denaturation steps, libraries were sequenced in a pool. A total of 50 ng of DNA was used to prepare the library, using the Nextera DNA sample preparation kit (Illumina). The library insert size was determined by an Experion automated electrophoresis station (Bio-Rad). The insert sizes of the libraries ranged from 300 to 850 bp (average, 500 bp). The library (12 pM) was loaded (in a pool) to a 600-cycle v3 reagent cartridge (Illumina) and the sequencing was performed on a MiSeq sequencer (Illumina) and *de novo* assembled with NGEN v. 11 (DNASTAR, Inc.). The genome was annotated using Prokka 1.7 (3).

The draft genome sequence of *Maribius* sp. strain MOLA401 is 3,856,666 bp, with a GC content of 67.6%, including 3,764 coding sequences, 1 rRNA, and 50 tRNAs. The genome annotation revealed the presence of genes involved in quorum sensing. The autoinducer type 1 synthase homologuous to *luxI*, and its corresponding receptor *luxR* (4) are located in the same operon. The genome annotation also revealed a gene encoding an RhtB-like protein, known to be involved in long-chain homoserinelactone transmembrane transport (5). This is the first report of quorum-sensing genes within the marine genus *Maribius*. Thus, this draft genome reinforces previous observations suggesting that marine bacteria are able to communicate using quorum sensing in microenvironments (6) where these cells can be found at high concentrations.

### Nucleotide sequence accession number.

The whole-genome shotgun project has been deposited at DDJB/EMBL/GenBank under the accession number JQEY00000000.
